# Adipocytes Protect Ovarian Cancer Cells from Ferroptosis

**DOI:** 10.3390/biom16101418

**Published:** 2026-09-30

**Authors:** Lia Tesfay, Chaeyeon Yoo, Timothy E. Moore, Jingyun Lee, Kirtikar Shukla, Cristina M. Furdui, Poornima Hegde, Evan R. Jellison, Suzy V. Torti

**Affiliations:** 1 Department of Molecular Biology and Biophysics, University of Connecticut Health Center, Farmington, CT 06030, USA; tesfay@uchc.edu; 2 Statistical Consulting Services, Center for Open Research Resources and Support, University of Connecticut, Storrs, CT 06269, USA; chaeyeon.yoo@uconn.edu (C.Y.); timothy.e.moore@uconn.edu (T.E.M.); 3 Department of Internal Medicine, Section on Molecular Medicine, Wake Forest University School of Medicine, Winston Salem, NC 27101, USA; jingyun.lee@advocatehealth.org (J.L.); kirtikar.shukla@advocatehealth.org (K.S.); cristina.furdui@advocatehealth.org (C.M.F.); 4 Department of Pathology and Laboratory Medicine, University of Connecticut Health Center, Farmington, CT 06030, USA; hegde@uchc.edu; 5 Department of Immunology, University of Connecticut Health Center, Farmington, CT 06030, USA; jellison@uchc.edu

**Keywords:** ferroptosis, lipid, adipocyte, ovarian cancer

## Abstract

Ferroptosis is a regulated form of cell death driven by lipid oxidation. Induction of ferroptosis has emerged as a potential approach for cancer treatment. Work published by our group and others has suggested that ovarian cancer may be among the potential clinical targets of ferroptosis inducers. The microenvironment plays a key role in all ovarian cancers, including high-grade serous ovarian cancer (HGSOC). In particular, the adipocyte-rich omentum is an important metastatic niche for ovarian cancer peritoneal metastases. Given the pivotal role of adipocytes in the ovarian cancer microenvironment, in this work we sought to understand the contribution of adipocytes to ovarian cancer ferroptosis. We report that adipocytes and the adipocyte-conditioned medium (ACM) protect ovarian cancer cells from ferroptosis through a multifactorial process involving changes in membrane lipid composition and lipid sequestration. Inhibiting lipid transfer significantly enhanced the anti-tumor effect of erastin, a ferroptosis inducer, in immune-deficient mice bearing intraperitoneal ovarian tumors. Disabling this cytoprotective pathway may be key to the effective deployment of ferroptosis inducers in the treatment of ovarian cancer.

## 1. Introduction

Ferroptosis is a regulated form of cell death driven by iron-dependent phospholipid peroxidation [1,2,3]. Ferroptosis is induced by drugs such as RSL3, which inhibits the activity of GPX4, an enzyme that reduces oxidized lipids [4]. Ferroptosis is also induced by compounds, like erastin [1], which inhibit the synthesis of glutathione, a GPX4 cofactor and cellular reductant. Induction of ferroptosis has emerged as a potential approach for cancer treatment [5,6]. Work published by our group and others has suggested that ovarian cancer may be among the potential clinical targets of ferroptosis inducers [7,8]. Ovarian cancer metastasis and drug resistance are believed to be driven by tumor-initiating cells with stem cell properties (“cancer stem cells”) [9,10]. Importantly, these cells are also susceptible to ferroptosis [7,10,11].

The microenvironment plays a key role in all ovarian cancers, including high-grade serous ovarian cancer (HGSOC), the most common and lethal subtype [12]. In particular, the adipocyte-rich omentum is an important metastatic niche for ovarian cancer peritoneal metastases [13]. The role of adipocytes in ovarian cancer is multifactorial [14]. Adipocytes secrete hormones, growth factors, cytokines, exosomal microRNA and other factors [13]. These impact chemotherapy resistance and also facilitate metastatic spread by promoting detachment, EMT, migration and invasion [13]. Additionally, adipocytes contribute to chemoresistance and metabolic reprogramming of ovarian cancer cells by releasing fatty acids [15] and enhancing fatty acid uptake in cancer cells through fatty acid chaperones such as FABP4 [16] and the fatty acid receptor CD36 [17].

Given the pivotal role of adipocytes in the ovarian cancer microenvironment, in this work we sought to understand the contribution of adipocytes to ovarian cancer ferroptosis. We report that lipid donation to ovarian cancer cells by adipocytes protects ovarian cancer cells from ferroptosis through a process involving changes in membrane lipid composition and lipid sequestration. Inhibiting lipid transfer significantly potentiated the anti-tumor effect of the ferroptosis inducer erastin in mice bearing peritoneal ovarian tumors. Disabling this cytoprotective pathway may be key to the effective deployment of ferroptosis inducers in the treatment of ovarian cancer.

## 2. Materials and Methods

### 2.1. Cell Culture 

OVCAR4 cells and OVCAR8 were obtained from the National Cancer Institute, Division of Cancer Treatment and Diagnosis (DCTD) Tumor Repository, Frederick National Laboratory for Cancer Research, Frederick MD on 25 February 2018, and cultured in RPMI 1640 + L-glutamine (GIBCO, Grand Island, NY, USA; cat# 11875-093; obtained from Fisher Scientific, Pittsburgh, PA, USA) supplemented with 10% FBS (Gemini Bio-Products, West Sacramento, CA, USA; Cat# 100-106). Cells were subcultured less than five times before use in experiments. FTT cells (described in [18]) were cultured in a DMEM medium (GIBCO; cat# 11965-095) containing 10% FBS. COV362 cells were obtained from Sigma (Cat# 07071910) and cultured in a DMEM medium (GIBCO; Ref# 11965-095) containing 10% FBS. All cells were maintained at 37 °C in a humidified incubator at 5% CO_2_.

### 2.2. Adipocyte Differentiation and Collection of Adipocyte-Conditioned Medium (ACM)

Human subcutaneous preadipocytes (HPAd-s) (ScienCell Research Laboratories, Carlsbad, CA, USA; cat# 802s-05a) were cultured in a preadipocyte growth medium (ScienCell Research Laboratories cat# 811-500) according to the supplier’s protocol and conditions. HPAd-s cells were allowed to differentiate for 15 days in a human adipocyte differentiation medium (Science Cell Research Laboratories Cat# 811D0250). Differentiated adipocytes (HAd) were incubated in a human adipocyte starvation medium (ScienCell Research Laboratories cat# 811S-250) for 24 h before replacing the medium with 10% FBS (Gemini Bio-Products) containing RPMI 1640 + L-Glutamine (GIBCO) or 10% FBS (Gemini Bio-Products) containing DMEM for 48 to 72 h to create an adipocyte-conditioned medium (ACM). ACM was removed with a pipet, leaving adherent adipocytes behind. The conditioned medium was stored at 4° until use. The control medium consisted of the same medium incubated under identical conditions in the absence of cells. 3T3-L1 mouse fibroblast cells were cultured in 10% FBS (Gemini Bio-Products) containing DMEM (GIBCO). 3T3-L1 mouse fibroblast cells were obtained from Wake Forest University Comprehensive Cancer Center Tissue Culture Core facility and grown in DMEM (GIBCO) plus 10% BCS (GE-lifesciences, Logan, UT, USA; cat# SH30072.03). When cells reached 70% confluence, they were incubated in a differentiation medium (D1) for 3 days (DMEM, 10% FBS, 0.5 mM IBMX (Sigma, St. Louis, MO, USA; Cat# 28822-58-4); 0.25 µM dexamethasone (Sigma; CAS# D4902), 10 µg/mL insulin (Sigma; CAS# 91077C)). Afterwards, the cells were further differentiated for 3 days with D2 medium (DMEM, 10% FBS, 10 µg/mL insulin (Sigma; CAS# 91077C)). Lastly, the fully differentiated 3T3-L1 adipocytes were kept in DMEM plus 10% FBS for 3 days. Human and mouse adipocytes were fully differentiated as measured by staining with Oil Red O (Sigma, CAS#1320-06-5).

### 2.3. Co-Culture Experiments 

3T3-L1 and HAd cells were fully differentiated before being overlaid with FTT cells and co-cultured for 24 h. Cells were treated with RSL3 (Selleckchem, Houston, TX, USA; Cat# S8155) in the presence or absence of ferrostatin-1 (Selleckchem; Cat# S7243) for 24 h. Cells were visualized with fluorescence microscopy and then subjected to FACS analysis.

### 2.4. Lipid Transfer

Human adipocytes (HAd) were fully differentiated before lipids were labeled with 5 µM BODIPY 558/568 C-12 (Invitrogen, Eugene, OR, USA; Cat# D3835) in adipocyte growth media for 4 h. Cells were then washed three times with 1XHBSS (GIBCO Ref# 14175-095) containing 0.2% fatty acid-free BSA (Sigma; Cat# A8806) before being overlaid with FTT cells for 24 h in the growth medium. Cells were fixed with 4% paraformaldehyde (Pierce, Rockford, IL, USA; cat# 28906) and mounted with Prolog (Invitrogen cat# P36980) before visualization by fluorescence microscopy (Zeiss, Jena, Germany).

### 2.5. Cell Viability and Treatments

Three to five × 10^3^ cells were seeded in 96-well plates and exposed to control or ACM for 24 h before treatment with the following reagents: RSL3 (Selleckchem; Cat# S8155), erastin (Selleckchem; Cat# S7242), ferrostatin-1 (Selleckchem; Cat# S7243), staurosporine (Sigma; Cat# 19-123), H2O2 (Sigma; Cat# 386790-M), DGAT1/2 inhibitors (Sigma; cat# T863 and PF06427878) at 20 µM, CD36 inhibitor sulfosuccinimidyl oleate sodium (SSO) (Cat #E2988, Selleckchem, Houston, TX, USA) at 100 µM, SCD1 inhibitors (MF-438 (Sigma; Cat# 569406)), CAY10566 (Cayman Chemicals, Ann Arbor, MI, USA: #10012562), A939572 (Cayman Chemical; Cat# 19123) at 1 µM, etoxomir (Selleckchem cat #S8244) at 40 µM. Cell viability was assessed 24–48 h post-treatment using calcein-AM (Invitrogen cat# C1430). Cell viability was also analyzed using Trypan-Blue-stain 0.4% (Invitrogen, Cat# T10282).

### 2.6. GSH/GSSG Assay

Cells were exposed to the control medium or ACM, lysed, and the GSH/GSSG ratio measured using the GSH/GSSG Glo (Promega, Madison, WI, USA; cat# V6611) kit according to the manufacturer’s protocol.

### 2.7. Spheroid Formation and Viability Assessment

A total of 8000 FTT cells were plated on round-bottom, ultra-low attachment 96-well plates (Nunc, obtained from Fisher Scientific), and allowed to form spheroids for 24 h followed by media change into conditioned media from adipocytes and incubated for 72 h. Cell viability was analyzed using ATP-based Celltiter Glo (Promega, G9241) 24 h after RSL3 treatment.

### 2.8. SiRNA Knockdown

A total of 12 ng of Silencer Select Human CD36 siRNA (Fisher Scientific; cat# 4392421), Silencer Select Human FAPB4 siRNA (Thermo Scientific; cat# 4392421) and control siRNA Silencer Select Negative Control siRNA (Thermo Scientific; 4390843) were used for knockdown experiments. Transfections were performed according to the manufacturer’s recommendations using Dharmafect #1 transfection reagent (Dharmacon; Horizon Discovery, Cambridge, UK; cat: T-2001). Knockdown efficiencies were confirmed at the time of harvest by RT-qPCR (Supplementary Materials Figure S1).

### 2.9. Real-Time RT-PCR

RNA was isolated using the High Pure RNA isolation kit (Roche Diagnostics, Indianapolis, IN, USA; Cat# 11828665001). A total of 200 ng of RNA was reverse-transcribed in a total volume of 50 μL using a reverse transcription reagents kit (Applied Biosystems, Carlsbad, CA, USA; cat#N8080234, obtained from Fisher Scientific). To make a standard curve, serial dilutions of RNA from one sample were added to the RT reaction. Aliquots (2 μL) of cDNA were added to an 18 μL reaction mixture containing 10 μL of 2× SYBR^®^ Green PCR Master Mix (BioRad, Hercules, CA, USA; cat# 1725124; obtained from Fisher Scientific) and 400 nm primers. Absence of DNA contamination was confirmed by performing PCR from cDNA without reverse transcriptase. Primers used were human FABP4 forward: ACG AGA GGA TGA TAA ACT GGT GG; human FABP4 reverse: GCG AAC TTC AGT CCA GGT CAA C; human CD36 forward: CAG GTC AAC CTA TTG GTC AAG CC; human CD36 reverse: GCC TTC TCA TCA CCA ATG GTC C; human Ferritin H (FTH) forward: CTT TGA CCG CGA TGA TGT GGC TTT; human Ferritin H (FTH) reverse: TTT GTC AGT GGC CAG TTT GTG CAG; human β-Actin forward: TTG CCG ACA GGA TGC AGA AGG A; and human β-Actin reverse: AGG TGG ACA GCG AGG CCA GGA T (all primers were from IDT).

### 2.10. Lipid Removal

Control and ACM were treated with a lipid removal agent (LRA) from Sigma/Millipore, St. Louis, MO, USA; (cat# 13358-U) at 4.3 mg/mL. Lipids bound to LRA were removed by centrifugation according to the manufacturer’s protocol. The centrifugation procedure was performed 3 times.

### 2.11. Lipid Droplet Staining

Cells were exposed to control or ACM in the presence or absence of DGAT1/2 inhibitors (Sigma cat# T863 and PF06427878) for 24 h. Cells were washed with 1XPBS, stained with 5µM BODIPY-493/503 (Invitrogen) for 30 min, washed and harvested for analysis by FACS or fixed with 4% paraformaldehyde (Pierce cat# 28906) and mounted using ProLong Gold with DAPI (Invitrogen cat #P36931) and visualized using confocal microscopy.

### 2.12. Lipid Peroxidation Staining

Cells exposed to control or ACM were treated with RSL3 (Selleckchem) before washing and staining with C11-bodipy (Invitrogen; cat# D3861) and analysis with a BD LSRII flow cytometer (Waters).

### 2.13. Western-Blot

Cells were lysed with RIPA buffer (Sigma-Aldrich, St. Louis, MO, USA; Cat# R0278) containing complete protease inhibitor cocktail (Roche Diagnostics; cat# 138468). Samples were separated by SDS–polyacrylamide gel electrophoresis and transferred to nitrocellulose membranes (BioRad, Hercules, CA, USA; cat# 162-0146) before being incubated with primary and secondary antibodies. Bands were detected using chemiluminescence (Pierce cat# 32106). Membranes were probed with antibodies to β-actin (Sigma; cat# A3854) and ferritin H [19].

### 2.14. Flow Cytometry

Single-cell suspensions were run on an LSRII flow cytometer (Waters). Flow Cytometry Standard (3.0) files were analyzed using FlowJo v10.10 (Waters) analysis software. Single cells and scatter gates were used to remove doublets and debris. Positive gates were drawn based on unstained or GFP negative controls, depending on the experiment.

### 2.15. Animal Experiments

Animal studies were conducted in accordance with the recommendations in the Guide for the Care and Use of Laboratory Animals of the Association for Assessment and Accreditation of Laboratory Animal Care International. The experimental protocol was approved by the Institutional Animal Care and Use Committee at the University of Connecticut Health Center (protocol # AP-201518). Female NOD.Cg-Prkdcscid Il2rgtm1Wjl/SzJ mice (NSG; ~6 weeks of age) were obtained from The Jackson Laboratory. FTT cells (100,000) were injected intraperitoneally (i.p.). Two experiments were performed, with 3 mice/group in the first experiment and 5 mice/group in the second. Sample size was based on prior experiments [7], with tumor number as the primary outcome measure. Mice were randomly allocated to control or treatment groups. No adjustment was made for potential confounders. Five days post-tumor implantation, mice were injected i.p. with 20 mg/kg CD36 inhibitor sulfosuccinimidyl oleate sodium (SSO) (Cat. #E2988, Selleckchem, Houston, TX, USA) in 5% DMSO (Fisher Chemicals, Pittsburgh, PA, USA; cat #D128-500) + 40% PEG300 (Selleckchem; cat #S6704) + 5% Tween80 (Selleckchem; cat #S6702) in the presence or absence of 20 mg/kg erastin (Selleckchem) in 5% DMSO + 40% PEG300 + 5% Tween80. Vehicle control was 5% DMSO + 40% PEG300 + 5% Tween80 in PBS (Gibco, cat. #10010-023). All solutions were made fresh prior to each use. Treatment was continued every other day for 15–17 days, at which time control mice became moribund. Mice were sacrificed, tumors were counted, and the combined weight of all tumors within the abdomen of each mouse was measured by an unblinded investigator. Mice were monitored daily for signs of distress and were weighed at the initiation of the study and every 5 days; mouse weights were not affected by treatments over the course of the experiment (Supplementary Materials Figure S2). One animal from the erastin group and another from the erastin + SSO group were found dead before the mice were sacrificed; these mice were removed from the analysis. Cause of death of these mice was not determined.

### 2.16. Statistical Analysis of Animal and Cell Culture Experiments

Statistical analyses were performed using GraphPad Prism 10 (version 10.4.1). All cell culture experiments were conducted at least 3 times using a minimum of three replicates per condition except the experiments shown in Figure 8, which were conducted twice. Statistical significance was assessed using t-tests and ANOVA. Statistics are reported as the mean ± standard deviation.

### 2.17. Fatty Acid Methyl Ester Analysis by GC-MS

*Extraction*—cell pellets were thawed and suspended in 1 mL of ice cold methanol, which were transferred to a homogenizer tube containing 0.5 mm glass beads. Cells were homogenized using a Bead Ruptor 24 (OMNI International, Kennesaw, Georgia) for 3 cycles of 20 s. The tubes were incubated on ice for 10 min and centrifuged for 10 min at 16,000× *g*. Supernatants were transferred into clean glass tubes, where 20 μL of an internal standard solution consisting of 0.8 μg/μL of 15:0 FAME and 0.2 μg/μL of 21:0 FAME was added. A total of 100 μL of 50% KOH was added and the extracts were blanketed with argon, mixed thoroughly and incubated at 60 °C for 1 h. The solution was extracted with 1 mL of water and 3 mL of hexane before acidification with 220 μL of 6 M HCl. Extraction with 3 mL of hexane was repeated, and the hexane layer collected and dried under nitrogen. Methylation was performed using 1 mL of 12% boron trifluoride in methanol under argon, mixing and incubating at 100 °C for 5 min. The solution was extracted with 2 mL of hexane and 4 mL of saturated NaCl, mixed thoroughly, and separated by centrifugation. The hexane layer was removed for analysis.

*Instrumental Analysis*—total fatty acid extracts were analyzed on a Trace Ultra gas chromatograph coupled to a TLQ Quantum XLS triple-quadrupole mass spectrometer (Thermo Scientific, Waltham, MA, USA). Separation was performed on a DB-WAXetr column (30 m × 0.25 mm, 0.25 µm film thickness, Agilent, Santa Clara, CA, USA) with a gradient temperature profile beginning with a 5 min hold at 100 °C followed by a 14 °C/min ramp to 200 °C, a 2 min hold at 200 °C, a 4 °C/min ramp to 230 °C, a 2 min hold at 230 °C, an 8 °C/min ramp to 260 °C, and, finally, a 10 min hold at 260 °C. The helium was used as carrier gas, the flow rate of which was 1.5 mL/min. Splitless injection was utilized for the first minute after injection at a temperature of 270 °C. The source was set to 200 °C and an electron energy of −42 eV. A full scan method from 50 to 450 *m*/*z* was utilized. Results presented are from duplicate biological cultures. Thermo Xcalibur Quan-Browser was used for quantitative data analysis.

### 2.18. Untargeted Lipidomics Analysis

*Extraction*—cells were thawed and suspended in 500 μL chilled methanol. The mixture was homogenized using 0.5 mm glass beads in a bead mill homogenizer for 3 × 20 s cycles with cooling between cycles. A total of 1 mL of chloroform was added, and the mixture was vortexed and incubated on ice for 30 min. Extracts were centrifuged at 16,000× *g* for 10 min, and the supernatant removed and dried under nitrogen. The residue was reconstituted in 100 μL of 1:1 isopropyl alcohol:methanol for analysis. *Instrumental Analysis*—samples were analyzed on a Q Exactive HF hybrid quadrupole-Orbitrap mass spectrometer and a Vanquish UHPLC system (Thermo Scientific, Waltham, MA, USA) coupled via heated electrospray ionization (HESI) source. Lipids were separated on an Accucore C30 column (2.6 µm, 3 mm × 150 mm, Thermo Scientific, Waltham, MA, USA) using a linear gradient with 60:40 acetonitrile/water (mobile phase A) and 90:10 isopropyl alcohol/acetonitrile (mobile phase B), both of which contain 0.1% formic acid and 10 mM ammonium formate. MS spectra were acquired by data-dependent scans in both positive and negative ion modes where the MS1 scan identifies the top ten most abundant precursor ions followed by MS2 (MS/MS) where product ions are generated from selected precursor ions. High-energy collisional dissociation (HCD) was applied for ion fragmentation with a stepped collision energy of 25/30 eV and 30/50/100 eV in positive and negative polarity mode, respectively. Dynamic exclusion was enabled during data-dependent scans. Data were searched using the LipidSearch v4.1.16 (Thermo Scientific, Waltham, MA, USA), the parameters of which were as follows: precursor mass tolerance, 5 ppm; product mass tolerance, 5 ppm; and phospholipid species selection, LPA, PA, LPC, PC, LPE, PE, LPG, PG, LPI, PI, LPS, PS, SM, MG, DG, TG, FA, CL, So, Cer, GMSGM1, GM2, CerG1, CerG2, CERG3, ChE, Co. Relative quantification was performed using peak areas that were normalized to the total ion current (TIC).

### 2.19. Lipidomics Statistical Analysis

Statistical analyses of lipidomic data were performed in R [20]. Prior to analysis, lipid abundance data were log2-transformed homoscedasticity. The comparison of the abundance between adipocyte-conditioned-medium-treated and control cells was assessed using the empirical Bayes-moderated t-test implemented in the *limma* package [21]. This approach is preferred over the standard two-sample t-test in small-replicate settings, as it borrows information across all 968 lipid species simultaneously to stabilize per-lipid variance estimates, leveraging the global variance structure to compensate for the limited sample size [22]. Lipids with a moderated *t*-test *p*-value below 0.05 were considered statistically significant. Results are displayed as volcano plots, where the *x*-axis represents the log_2_ fold change between conditions, reflecting the magnitude and direction of lipid abundance change, and the *y*-axis represents the −log_10_ *p*-value, reflecting statistical significance at α = 0.05. Principal component analysis (PCA) was performed using Lipidr [23].

## 3. Results

### 3.1. Adipocytes and the Adipocyte-Conditioned Medium (ACM) Protect Ovarian Cancer Cells from Ferroptosis but Not Apoptosis or Necrosis

**Figure 1:**
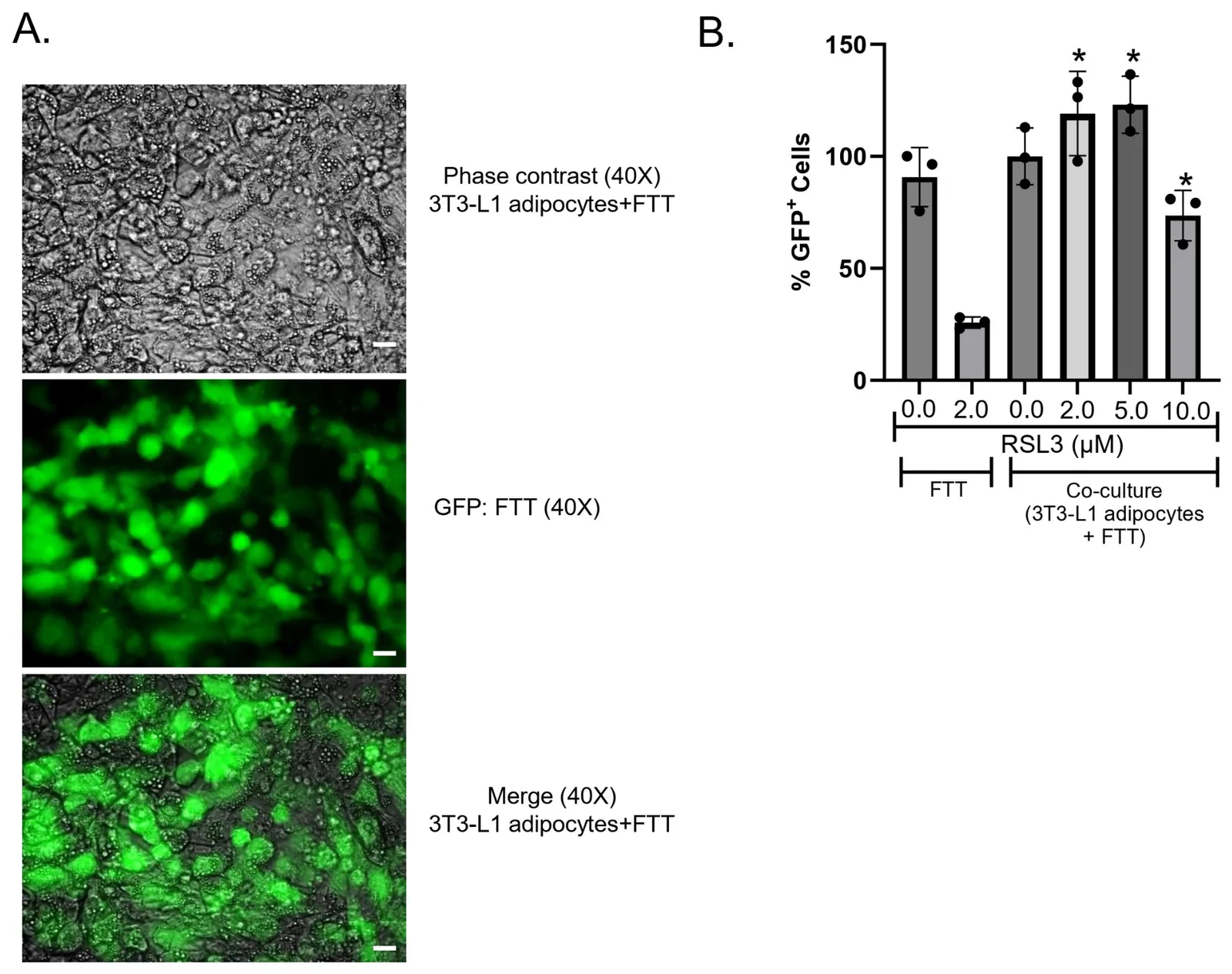
Co-culture of ovarian cancer cells with 3T3-L1 adipocytes protects cancer cells from ferroptosis: FTT cells were overlaid on a confluent monolayer of 3T3-L1 adipocytes for 24 h, then treated with RSL3 for 24 h. (**A**) Phase contrast and fluorescence images of a representative field of cells; (**B**) GFP-positive FTT cells were quantified by FACS analysis. Shown are means and stdev of 3 replicates; results typical of 3 independent experiments. Comparisons of FTT treated with RSL3 to FTT co-cultured with adipocytes and treated with RSL3 Scale bar 10 µM; * *p* < 0.05 (*t*-test).

To test whether adipocytes affect the response of ovarian cancer cells to ferroptosis, we first examined GFP-labeled FTT cells, a model of ovarian cancer stem cells [18]. We plated these ovarian cancer cells on a layer of differentiated 3T3-L1 murine adipocytes (Figure 1A) and measured the response of the cancer cells to the ferroptosis inducer RSL3. As shown in Figure 1B, co-culture with adipocytes significantly protected these ovarian cancer stem-like cells from the cytotoxic effects of RSL3. Lipid peroxidation, a marker of ferroptosis, was also reduced (Supplementary Materials Figure S3).

**Figure 2:**
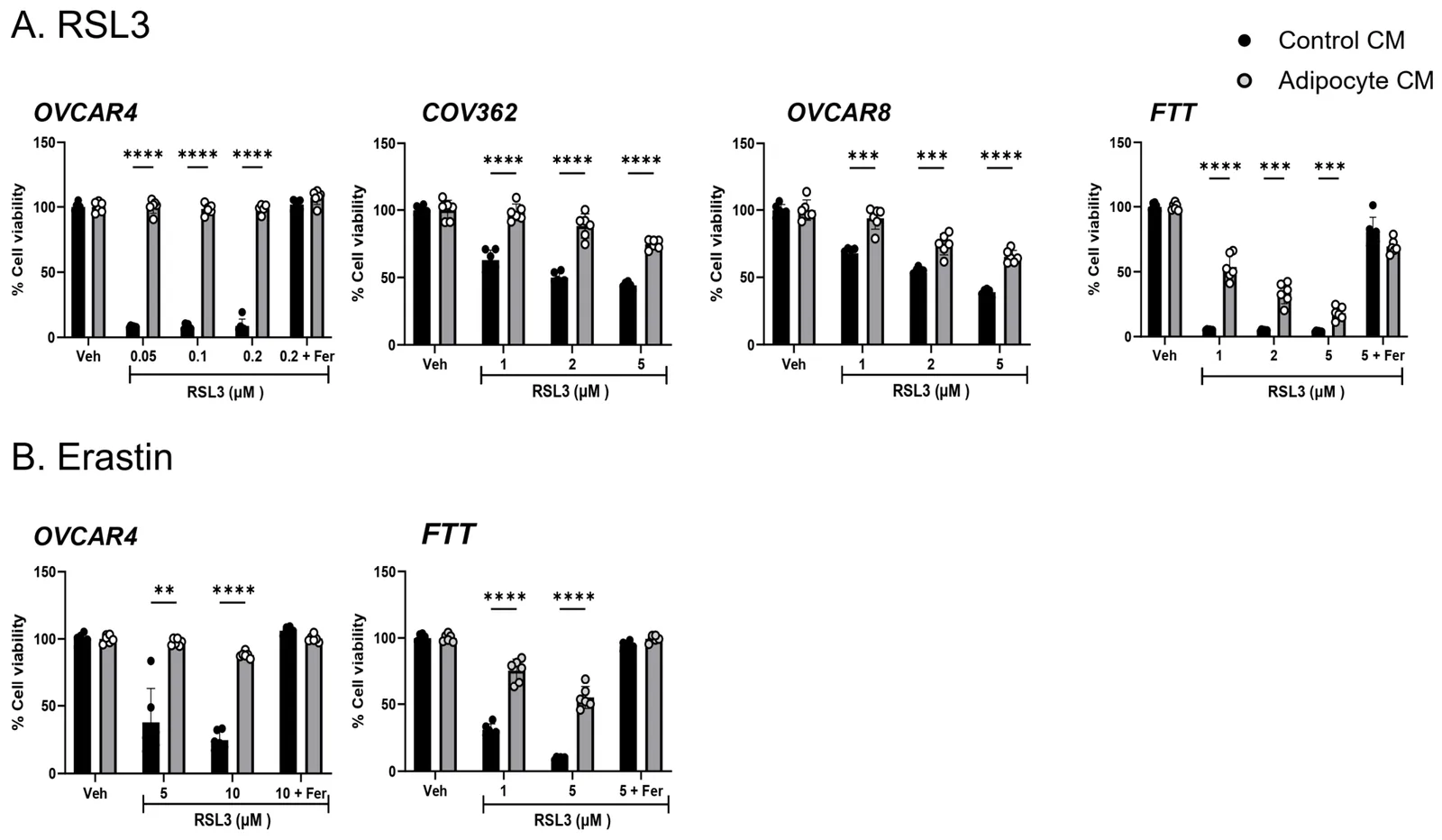
Conditioned medium from human adipocytes (ACM) protects multiple ovarian cancer cell lines from ferroptosis. (**A**) ACM was applied to ovarian cancer cell lines for 24 h prior to treatment with vehicle (Veh), the ferroptosis inducer RSL3, RSL3 + the ferroptosis inhibitor ferrostatin (Fer), and cell viability assessed; (**B**) Cells were treated as in (**A**) except that erastin was used in place of RSL3. Means and stdev of 6 replicates; results typical of 3 independent expts. For COV362 and OVCAR8 means and stdev of 6 replicates; results typical of 2 independent expts. ** *p* < 0.01, *** *p* < 0.001, **** *p* < 0.0001 (2-way ANOVA).

To determine whether the protective effect on ferroptosis required direct cell contact, we tested whether similar protection could be conferred by the adipocyte-conditioned medium (ACM). We also tested whether the protective effect of adipocytes would extend to human HGSOC cell lines. For these experiments we used a conditioned medium from human adipocytes that had been differentiated from preadipocytes. As shown in Figure 2A, in both FTT and OVCAR4 cells, ACM was effective in preventing cell death induced by RSL3. Cell death was blocked by the ferroptosis inhibitor ferrostatin, confirming ferroptosis as the mechanism of cell death (Figure 2A). Ferroptosis was similarly reduced by ACM in OVCAR8 and COV362, two other HGSOC cell lines (Figure 2A). To support these results, we assessed the effect of ACM on the response to erastin, a mechanistically dissimilar ferroptosis inducer. Both OVCAR4 and FTT cells were similarly protected from erastin by ACM (Figure 2B). ACM did not affect proliferation (Supplementary Materials Figure S4).

**Figure 3:**
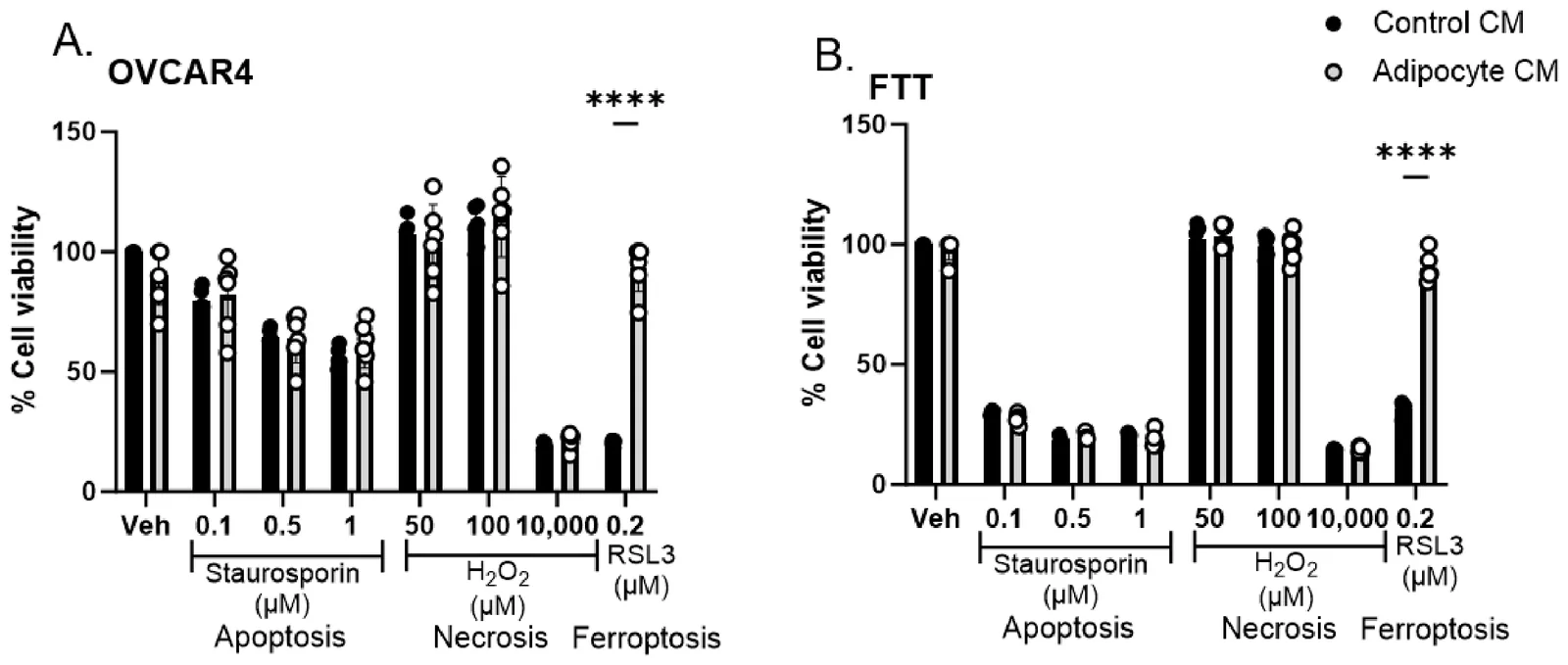
Conditioned medium from human adipocytes does not protect ovarian cancer cells from apoptosis or necrosis. (**A**) ACM was applied to OVCAR4 ovarian cancer cells for 24 h prior to treatment with vehicle (Veh), staurosporin, H2O2, or RSL3, and cell viability assessed using calcein-AM; (**B**) The same treatments were applied to FTT cells. Means and stdev of 6 replicates; results typical of 3 independent experiments. **** *p* < 0.0001 (2-way ANOVA).

To assess the specificity of this protective mechanism, we tested whether ACM would similarly protect ovarian cancer cells from inducers of apoptosis and necrosis. To induce apoptosis, we challenged cells with staurosporine; necrosis was induced by exposing cells to hydrogen peroxide [24]. As shown in Figure 3, ACM had no effect on cell death triggered by either of these inducers, suggesting a preferential effect of adipocytes on prevention of ferroptotic cell death.

### 3.2. Spheroids Are Also Protected from Ferroptosis by ACM

Ovarian cancer is thought to disseminate in the peritoneal cavity as groups of cells assembled into three-dimensional spheroidal metastatic units [25]. To test the role of adipocytes in modulating the ferroptosis of these metastatic units, we first created ovarian cancer spheroids and tested their sensitivity to RSL3. As shown in Supplementary Materials Figure S5, ovarian cancer cells organized as three-dimensional spheroids retained their sensitivity to the ferroptosis inducers RSL3 and erastin.

**Figure 4:**
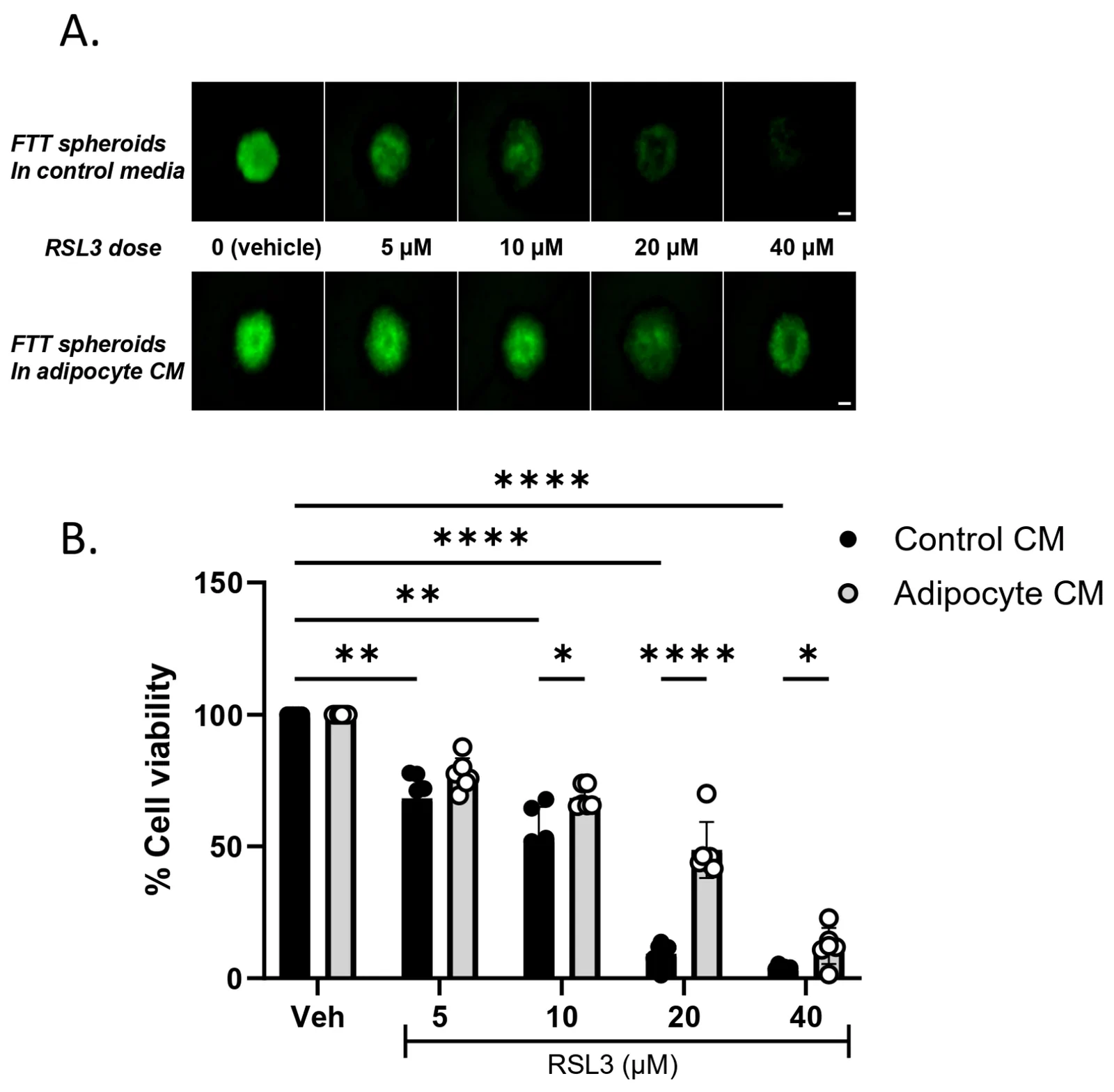
ACM protects ovarian cancer spheroids from ferroptosis. FTT cells were allowed to form spheroids for 24 h followed by exposure to control or adipocyte-conditioned media for 72 h and subsequent treatment with RSL3. (**A**) Representative image of spheroids: (**B**) Cell viability was analyzed using ATP-based Celltiter Glo 24 h after RSL3 treatment. Means and std dev of 3 to 8 replicate samples; one of three independent expts. * *p* < 0.05; ** *p* < 0.01; **** *p* < 0.0001 (2-way ANOVA). Scale bar: 100 µm.

We then assessed the effect of exposing these spheroids to ACM prior to treatment with RSL3. As shown in Figure 4, ACM reduced the cytotoxic effect of RSL3 on ovarian cancer spheroids as well.

### 3.3. Exposure to ACM Does Not Protect Ovarian Cancer Cells from Ferroptosis by Increasing the Ratio of Reduced to Oxidized Glutathione or Energy Production

We next sought to understand the mechanism(s) underlying the cytoprotective effect of ACM on ferroptosis.

**Figure 5:**
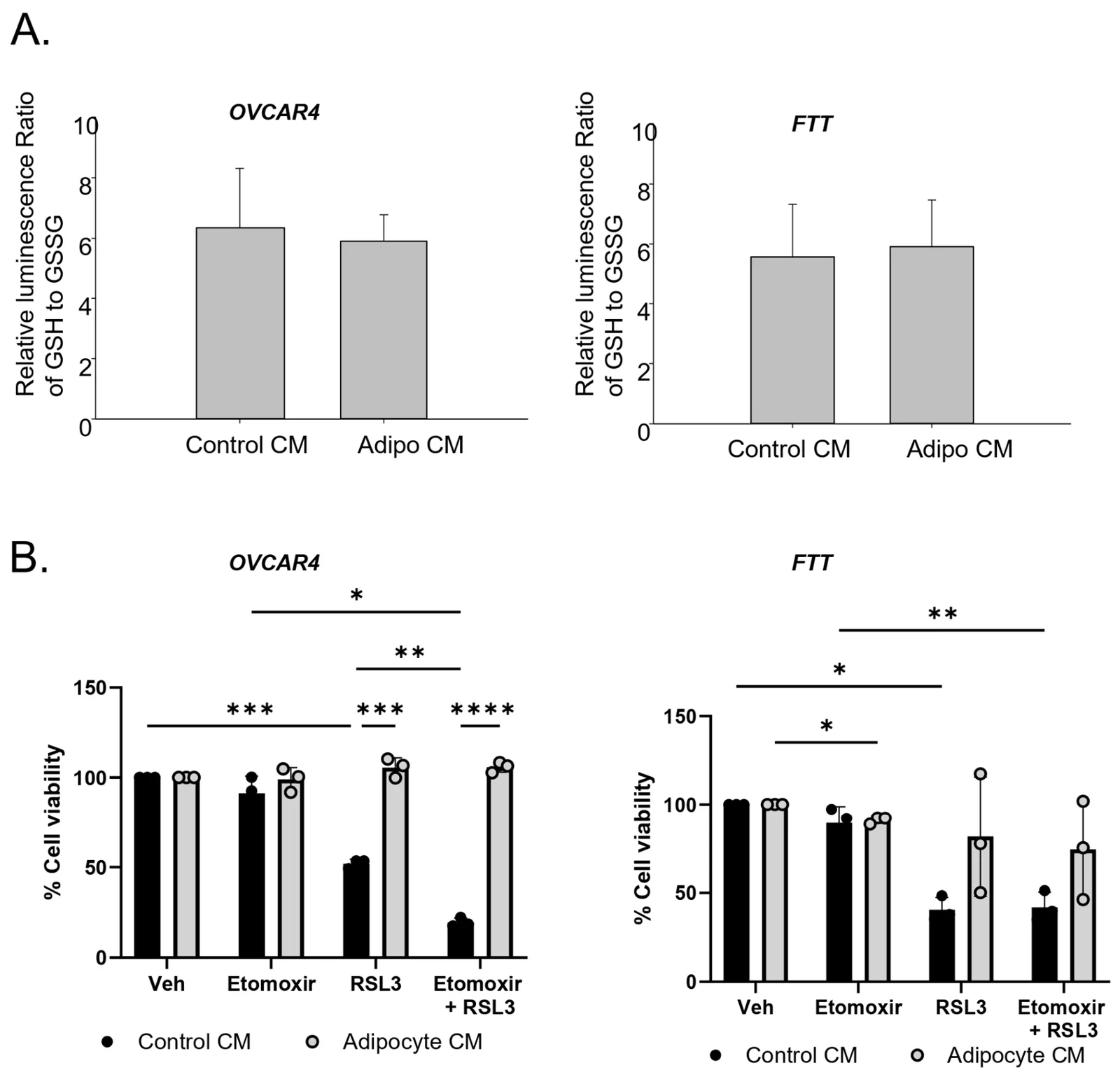
The protective effect of ACM is independent of the ratio of reduced to oxidized glutathione or beta oxidation. (**A**) The ratio of reduced/oxidized glutathione (GSH/GSSG) remains unchanged in ovarian cancer cells exposed to control or ACM. Ovarian cancer cells were incubated with control or ACM for 48 h. Cells were then subjected to GSH/GSSG-Glo assay. Shown are means and std dev of 3 independent experiments with six replicates per experiment (ns, *t* test). (**B**) Etomoxir does not block the cytoprotective effect of ACM on RSL3-mediated ferroptosis. Adipocyte-conditioned or control medium was applied to ovarian cancer cells in the presence of etomoxir for 24 h prior to treatment with vehicle (Veh), the ferroptosis inducer RSL3 (0.2 µM OVCAR4; 2 µM FTT) and cell viability was assessed; shown are means and std dev of 3 independent experiments, with six replicates per experiment. * *p* < 0.05; ** *p* < 0.01, *** *p* < 0.001, **** *p* < 0.0001, 2-way ANOVA.

We first explored the effect of ACM on the level of reduced glutathione in ovarian cancer cells. Adipocytes have been shown to provide reducing equivalents to cancer cells through activation of the TCA cycle [26]. Since GSH is required for the activity of GPX4, an increase in the GSH/GSSG ratio would be expected to protect cells from ferroptosis. However, as shown in Figure 5A, ACM did not appreciably affect the GSH/GSSG ratio in either OVCAR4 or FTT cells.

Lipids and more particularly lipid peroxidation are central features of ferroptosis. Degradation of lipids by beta oxidation is used by cells to provide energy. It has been previously shown that inhibiting beta oxidation potentiates ferroptosis, possibly by preserving lipid substrates for the peroxidation required for ferroptosis [27]. Since adipocytes provide substrates for fatty acid oxidation to ovarian cancer cells [26], we tested whether ACM might exert its protective effect through a stimulatory effect on beta oxidation. To examine this possibility, we treated cells with etomoxir, an inhibitor of CPT1, the rate-limiting step of the CPT system [28]. Treatment with etomoxir did not substantially reduce the cytoprotective effect of ACM on ferroptosis (Figure 5B), suggesting that stimulation of beta oxidation does not underlie the cytoprotective effect of ACM on ferroptosis. In the case of OVCAR4 cells, we also noted that in the absence of ACM, inhibiting beta oxidation with etomoxir potentiated RSL3-mediated ferroptosis, consistent with prior results [29,30].

### 3.4. Transfer of Lipids from Adipocytes to Ovarian Cancer Cells Plays a Major Role in Protection from Ferroptosis

**Figure 6:**
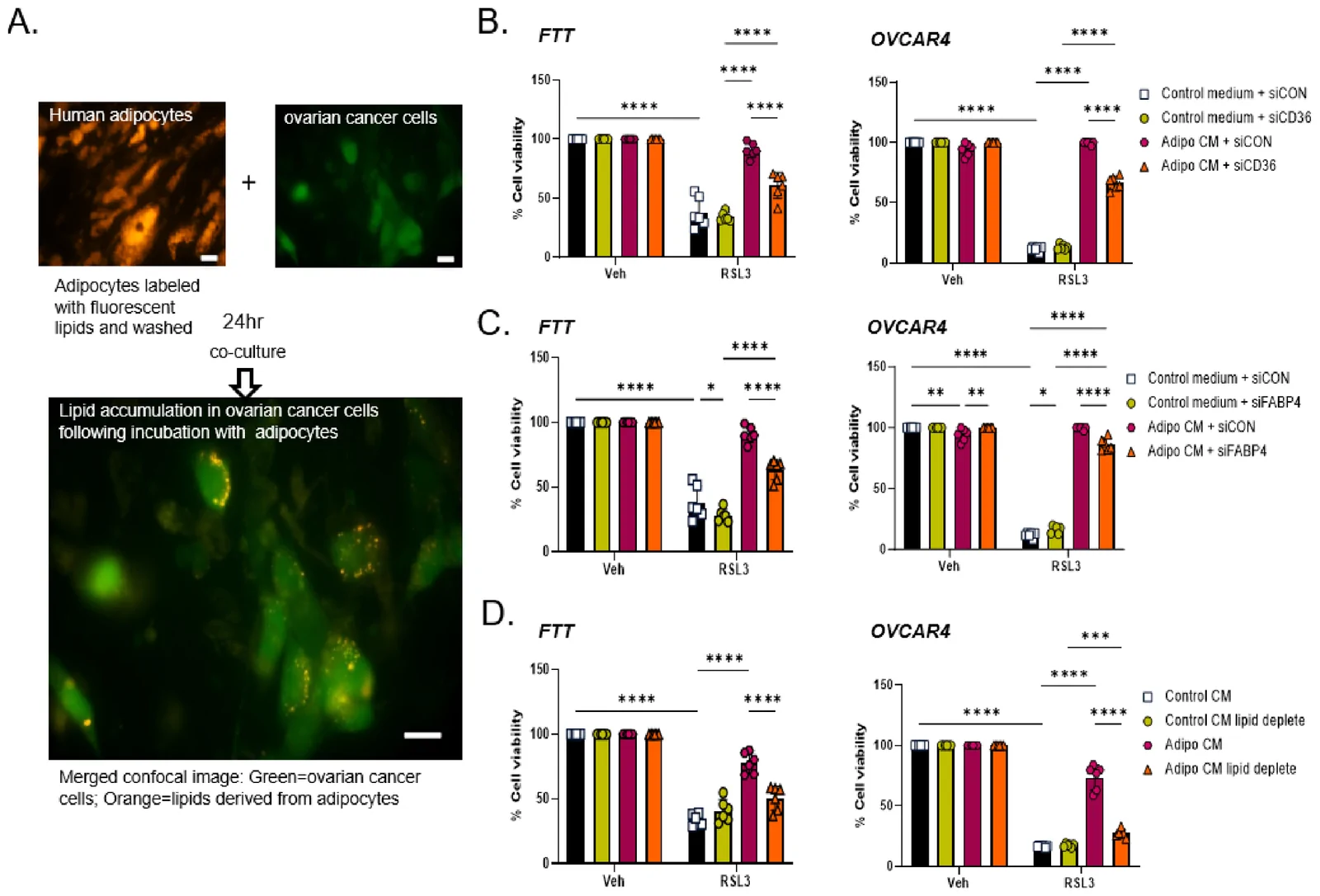
Inhibiting lipid uptake reduces the cytoprotective effect of ACM on RSL3-dependent ferroptosis. (**A**) Ovarian cancer cells (OC) take up lipids from adipocytes. Adipocytes were labeled with the lipid specific fluorescent dye (Fluoro-Lipids/BODIPY C12), washed, and overlaid with EGFP-expressing ovarian cancer cells (FT-T). Fluorescence imaging was performed 24 h later. (**B**,**C**) Inhibiting lipid uptake pathways reduces the cytoprotective effect of ACM. Ovarian cancer cells (OVCAR4) were transfected with control or CD36 siRNA or FABP4 siRNA for 72 h before ACM or control medium was applied for 24 h. Cells were then treated with RSL3. Cell viability was assessed using calcein-AM. Means and stdev of 6 replicates; results typical of 3 independent expts. (**D**) Depleting lipids from ACM reduces its protective effect. Control and adipocyte-conditioned media were treated with lipid removal agent (LRA) prior to treatment with RSL3 and measurement of viability. Scale bar is 20 µM. Means and stdev of 6 replicates; results typical of 3 independent expts * *p* < 0.05; ** *p* < 0.01, *** *p* < 0.001, **** *p* < 0.0001, 2-way ANOVA.

Adipocytes secrete multiple bioactive molecules, including adipokines, cytokines, exosomes and fatty acids [31]. We tested whether the cytoprotective effect of ACM requires the presence of lipids in the media and the subsequent transfer of these lipids to ovarian cancer cells. We first confirmed that ovarian cancer cells could acquire lipids from adipocytes. We labeled adipocytes with BODIPY C12, a fluorescent long-chain fatty acid analog that is incorporated into multiple lipid species [32,33]. Adipocytes were then washed and overlaid with EGFP-tagged ovarian cancer cells prior to treatment with RSL3. As shown in Figure 6A, consistent with previous reports [34], labeled lipids were transferred from adipocytes to ovarian cancer cells under these conditions.

Ovarian cancer cells take up lipids using a variety of mechanisms. Prominent among these is the fatty acid receptor CD36, which is induced by adipocytes in ovarian cancer [35] and has been reported to play a role in the metastatic spread of ovarian cancer [17]. To confirm the requirement for lipid uptake in the cytoprotective effect of adipocytes, we therefore used siRNA to knock down CD36 in OVCAR4 cells and then assessed the ability of cells to be protected from ferroptosis by a conditioned medium from adipocytes. As shown in Figure 6B, knockdown of CD36 significantly diminished the ability of the conditioned medium to protect ovarian cancer cells from the ferroptosis inducer RSL3, consistent with a protective mechanism mediated by lipid uptake. Knockdown of the lipid chaperone FABP4 also reduced the cytoprotective effect of ACM on ferroptosis, although in the case of OVCAR4 cells, the effects of FABP4 were more modest (Figure 6C).

To corroborate these findings, we depleted ACM of lipids using a lipid removal agent (LRA), a synthetic lipid adsorbent. Lipid depletion did not affect viability (Supplementary Materials Figure S6). As shown in Figure 6D, a conditioned medium that had been lipid depleted was significantly less effective in conferring protection from ferroptosis than an undepleted medium. Thus, lipid transfer from adipocytes to ovarian cancer cells makes a major contribution to the protective effect of adipocytes.

### 3.5. Ovarian Cancer Cells Exposed to ACM Exhibit Significant Qualitative Changes in Lipid Composition

**Figure 7:**
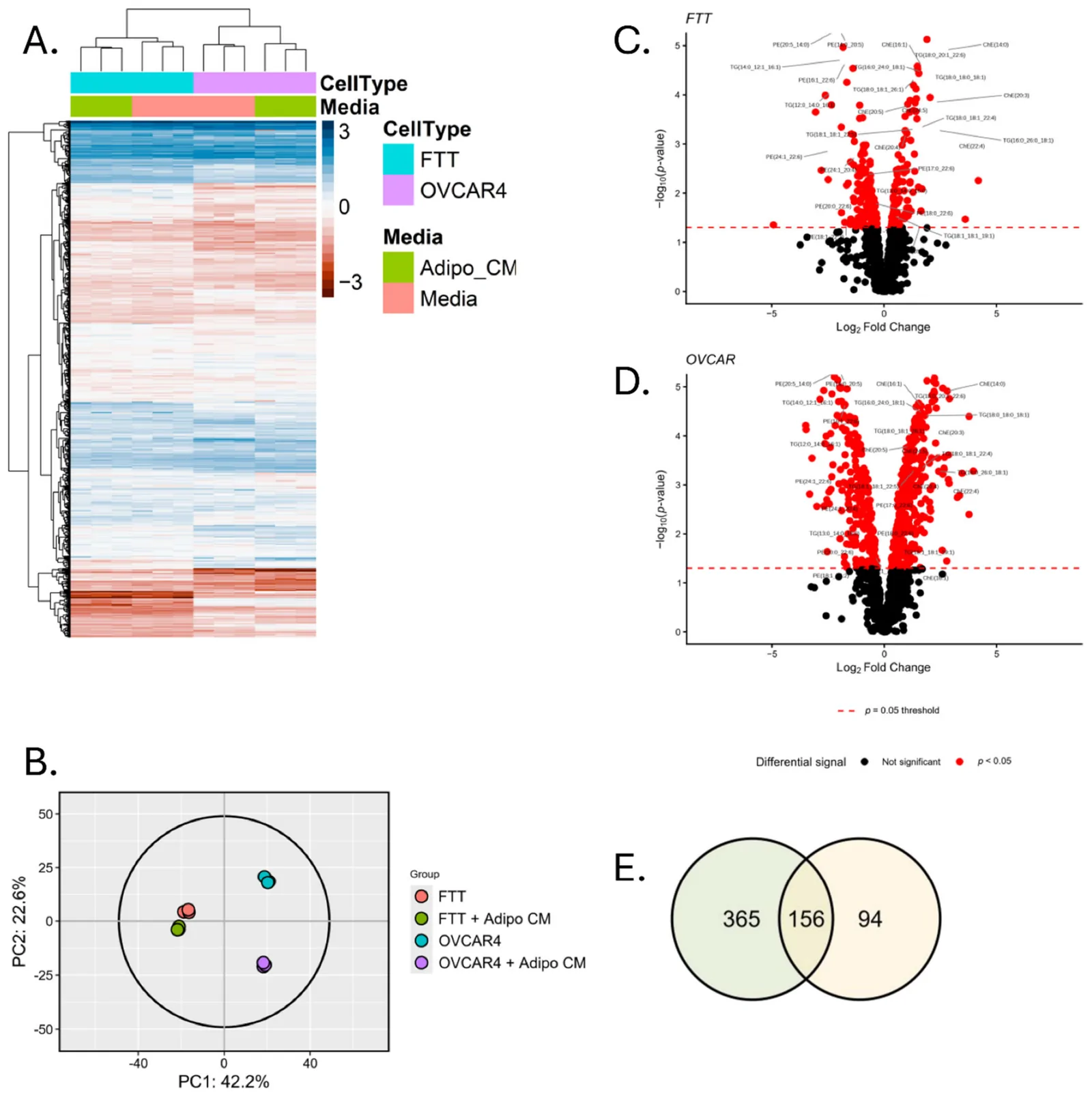
Lipidomic analysis reveals common and cell line-dependent alterations in lipid profiles induced by exposure to ACM. (**A**) Heatmap of scaled log2 values according to cell type and media. All cells have a mean of 0 and standard deviation of 1. (**B**) Principal component analysis (PCA) shows distinct clustering of experimental groups with tight grouping of biological replicates, indicating high reproducibility and group-specific molecular profiles. (**C**,**D**) Volcano plots of lipids; selected lipids are noted. (**E**) Venn diagram of the number of lipid species with significant changes following exposure to ACM in OVCAR4 and FTT cells.

To assess the impact of ACM on the lipid composition of ovarian cancer cells, we performed a lipidomics analysis of OVCAR4 and FTT cells that had either been exposed to a basal medium or ACM. The results of this nontargeted analysis of 968 lipid species demonstrated an impact of exposure to ACM on lipid composition in both cells (Figure 7). Principal component analysis (PCA) revealed distinct global lipid signatures in each group, with tight grouping of replicates within each condition, minimal within-group variability, and strong between-group differences (Figure 7B). Absent exposure to ACM, there was a notable difference in baseline lipid composition between OVCAR4 and FTT cells (Figure 7A,B). ACM affected lipid profiles in both cells, but the extent of the effect differed between the two cell types: lipid profiles of control and ACM-treated FTT cells (Figure 7B, red and green groups respectively) clustered closely, suggesting distinct but similar overall profiles, whereas lipid profiles of control and ACM-treated OVCAR4 cells (cyan and purple respectively) groups varied more widely, indicating a greater impact of ACM on overall lipid profile. Specifically, 521 lipid species were significantly altered by ACM in OVCAR4 cells, whereas 250 lipid species were altered in FTT cells (Figure 7C,D; Supplementary Materials Table S1). Changes were observed in all lipid classes, including ceramides, cholesterol esters, diacylglycerides, phosphatidyl choline, phosphatidylethanolamine, phosphatidyl inositide, phosphatidyl serine, plasmalogens, and triglycerides (Supplementary Materials Table S1).

Of the 968 lipids profiled, 156 were significantly altered in both cell lines, while 365 were significant in OVCAR4 only and 94 in FTT only (Figure 7E). Within the group of 156 lipid species altered in both cell lines, we focused our attention on phosphatidylethanolamines, since although PUFAs found in multiple phospholipids contribute to ferroptosis [3], peroxidation of PUFA fatty acids linked to PE has been highlighted as a major feature of ferroptosis [36]. Out of 12 species of PUFA-containing PE species, 10 decreased in both cell types following exposure to ACM, a change consistent with reduced susceptibility to ferroptosis. We also examined effects of ACM on CoQ, a lipophilic endogenous antioxidant that confers protection to ferroptosis [37]. Consistent with increased resistance to ferroptosis, levels of CoQ(9) were significantly increased. Levels of CoQ(10) also exhibited a trend towards an increase, although differences were not statistically significant. Among other lipids, eight species of cholesterol esters significantly increased in both cell types following exposure to ACM and 27 triglyceride species were significantly changed in both cells, with the majority (18) increased and the remainder decreased following treatment with ACM.

### 3.6. Exposure to ACM Promotes Sequestration of Lipids in Lipid Droplets

**Figure 8:**
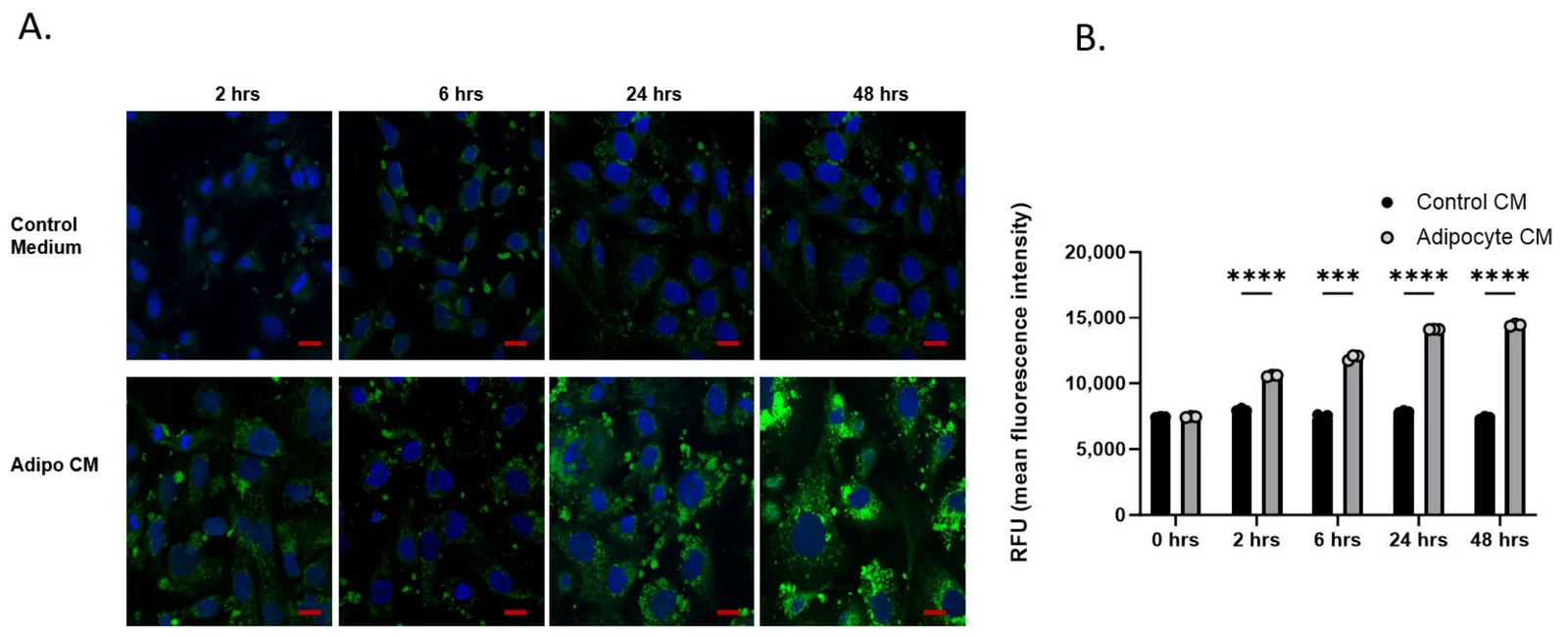
ACM promotes the sequestration of lipids in lipid droplets. OVCAR4 cells were exposed to control or ACM for 2–48 h before staining lipid droplets with BODIPY-493/503. (**A**) Representative image of cells visualized with fluorescence microscopy; blue, DAPI; green, lipid droplets. (**B**) Quantification of fluorescence by flow cytometry. Means and SD of data from 2 independent experiments with ≥3 replicas per experiment. Scale bar: 10 µm. *** *p* < 0.001, **** *p* < 0.0001, 2-way ANOVA.

Sequestration of lipids in lipid droplets is a mechanism that reduces lipid susceptibility to peroxidation [38]. Given the increased levels of cholesterol esters and triglycerides with ACM exposure, we tested whether there was an accumulation of lipid droplets following exposure to ACM. Lipid droplets were visualized in cells before and after exposure to ACM using BODIPY 493/503. As shown in Figure 8, lipid droplet accumulation was significantly increased in ovarian cancer cells following exposure to ACM.

### 3.7. The ACM-Dependent Increase in Lipid Droplets Contributes to Protection from Ferroptosis

**Figure 9:**
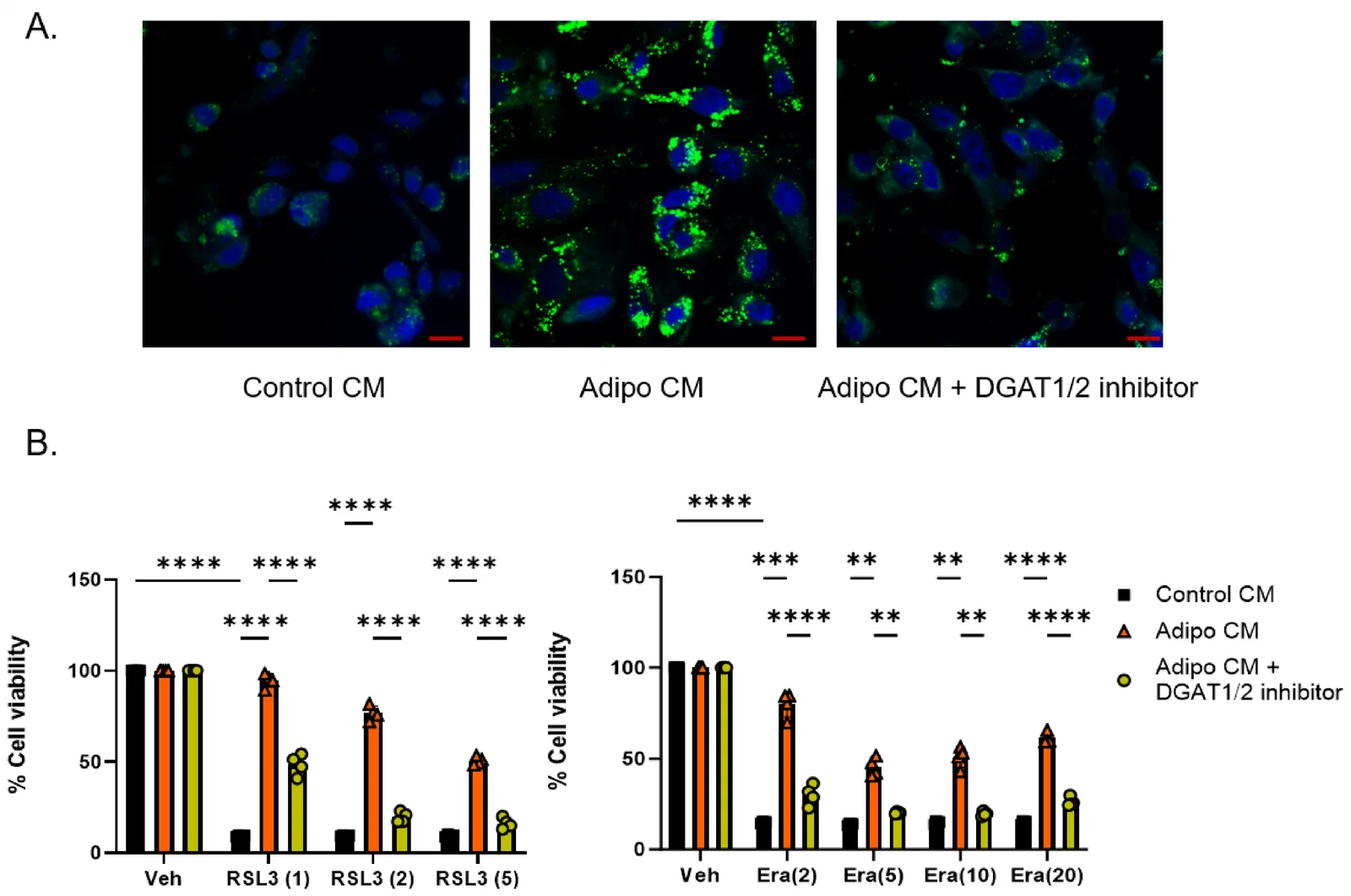
DGAT 1/2 inhibitors reduce lipid droplets in cells exposed to ACM and block the protective effect of ACM. (**A**) OVCAR4 cells were exposed to control or ACM in the presence or absence of DGAT1/2 inhibitors for 24 h. Lipid droplets were stained using BODIPY 493/503 and visualized using confocal microscopy. Blue, DAPI; green, lipid droplets. Scale bar 10 µm. (**B**) OVCAR4 cells were exposed to control or ACM in the presence or absence of DGAT1/2 inhibitors followed by RSL3 or erastin treatment and assessment of cell viability. Shown are means and std dev of 3 independent experiments, with six replicates per experiment, ** *p* < 0.01, *** *p* < 0.001, **** *p* < 0.0001, 2-way ANOVA.

Lipid droplet formation is mediated by diacylglycerol acyltransferases DGAT1 and DGAT2, which catalyze the last step in diacylglycerol synthesis by adding a fatty acyl group to the *sn*-3 position of diacylglyerol [39,40]. To test whether lipid droplet accumulation contributed to the protective effect of ACM on ferroptosis, we blocked these enzymes using the DGAT1 and DGAT2 inhibitors T863 and PF-06424439. As shown in Figure 9, pre-treatment with these inhibitors reduced both lipid droplets and the protective effect of ACM on ferroptosis induced by either RSL3 or erastin.

### 3.8. Inhibiting SCD1 Attenuates the Protective Effect of ACM

**Figure 10:**
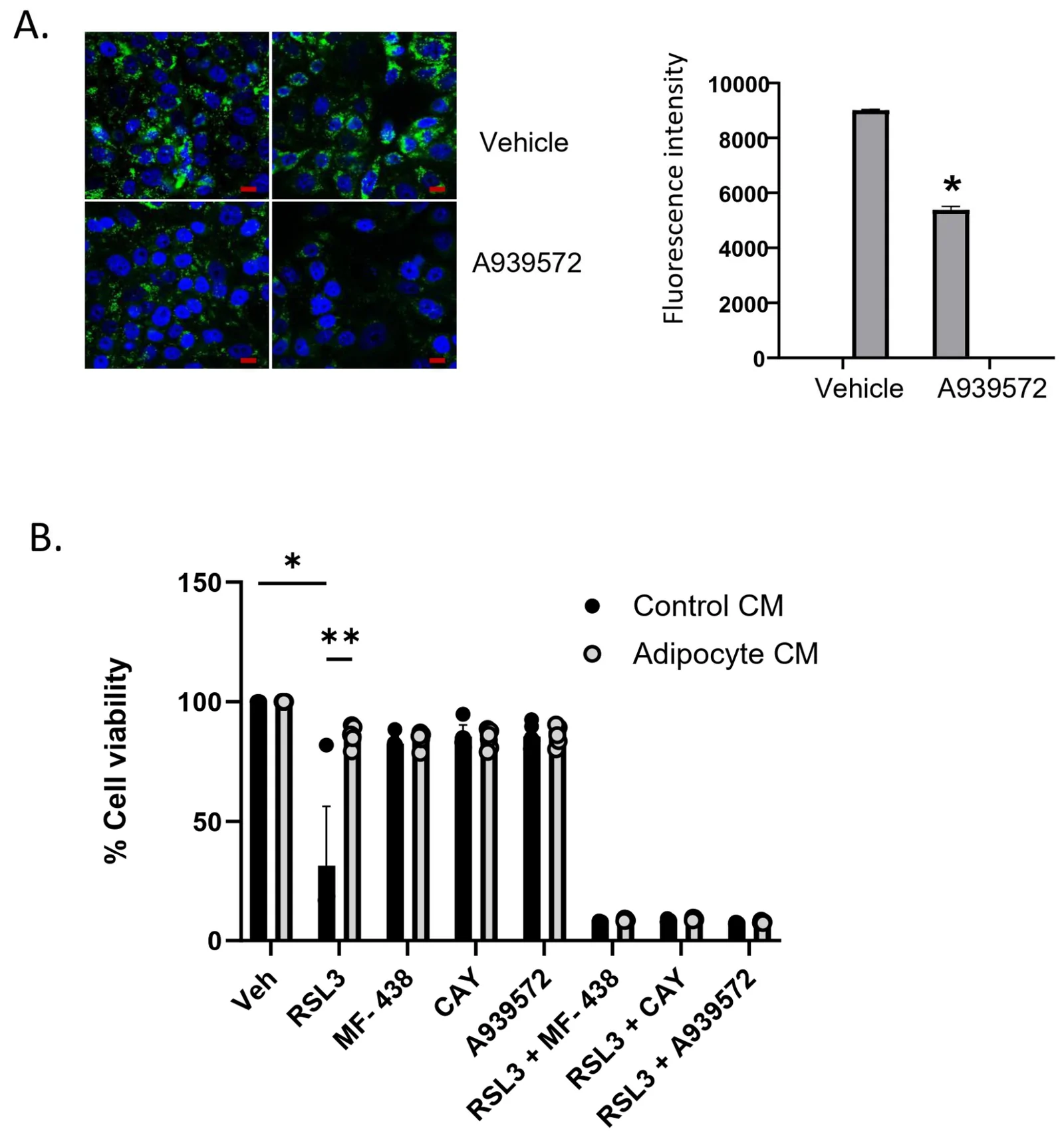
Inhibition of SCD1 reduces lipid droplets and attenuates the cytoprotective effect of ACM on ferroptosis. (**A**) SCD1 inhibitor reduces lipid droplets. OVCAR4 cells were exposed to ACM in the presence or absence of the SCD1 inhibitor A939572 for 24 h and then stained with BODIPY 493/503. Cells were imaged (left) and fluorescence intensity quantified using FACS (*t*-test); blue, DAPI; green, lipid droplets. (**B**) Multiple SCD1 inhibitors block the cytoprotective effect of ACM in cells exposed to RSL3. Scale bar: 10 µm. * *p* < 0.05; ** *p* < 0.01, 2-way ANOVA.

We have previously observed that stearoyl-CoA desaturase 1 (SCD1), an enzyme that catalyzes the rate-limiting step in monounsaturated fatty acid synthesis, protects ovarian cancer cells from ferroptotic cell death, in part by maintaining levels of monounsaturated fatty acids and also by increasing CoQ [41]. SCD1 also contributes to lipid droplet formation [42], and we reasoned that this might represent an additional mechanism by which SCD1 protects against ferroptosis, particularly in cells exposed to ACM. To test this possibility, we assessed the effect of inhibiting SCD1 on the protective effect of ACM. As shown in Figure 10, treatment with SCD1 inhibitors dramatically attenuated both lipid droplet content and the cytoprotective effect of ACM on ferroptosis.

### 3.9. Inhibiting Lipid Uptake Sensitizes Ovarian Cancer Cells to Ferroptosis In Vivo

**Figure 11:**
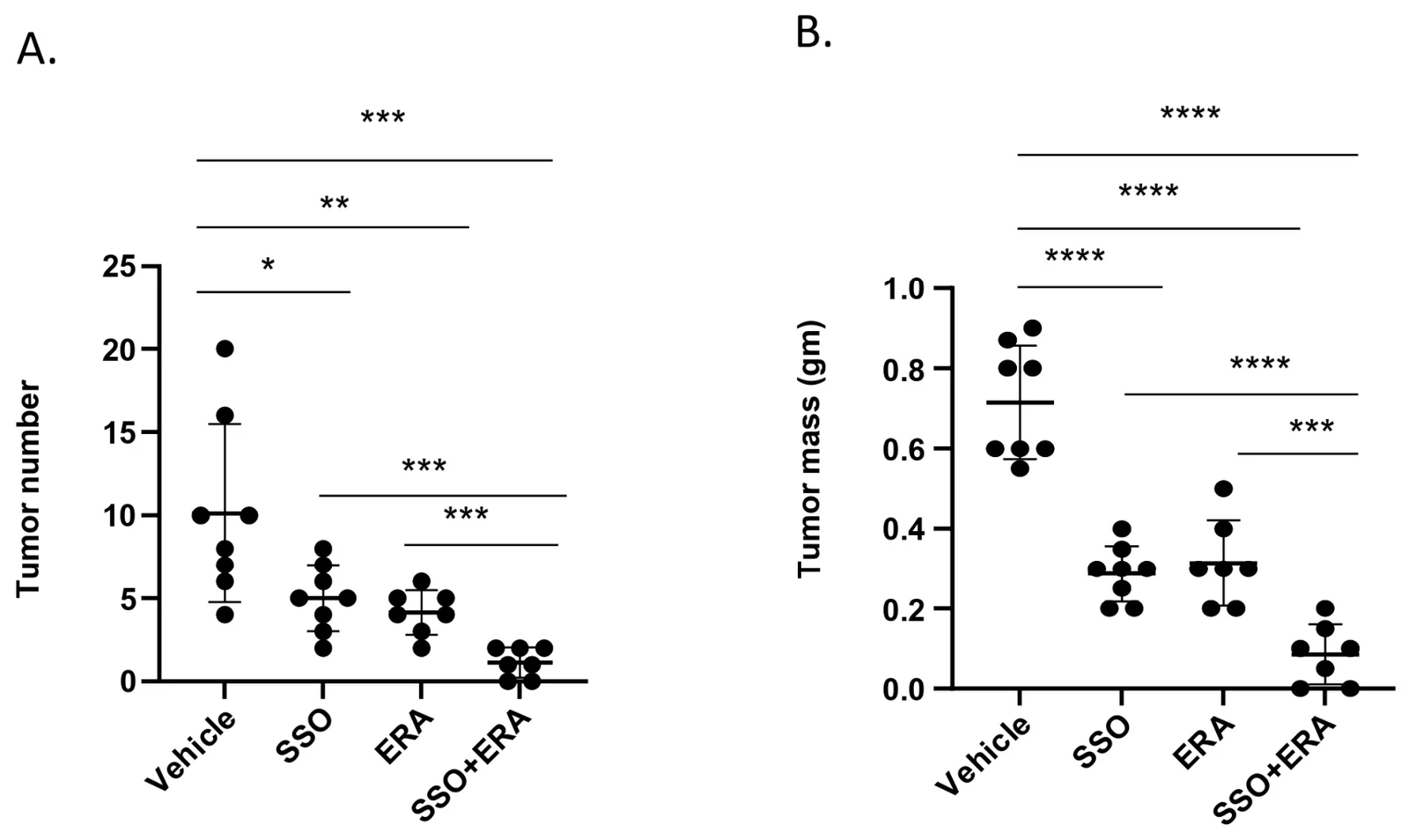
Inhibition of CD36 enhances the anti-tumor efficacy of a ferroptosis inducer in vivo. Tumor-bearing mice were treated with vehicle, the CD36 inhibitor SSO, erastin, or the combination of SSO and erastin. The experiment was performed twice; the combined results of two experiments are shown (total of *n* = 8/group). (**A**)Tumor number and (**B**) total tumor mass were quantified after 15–17 days. * *p* < 0.05; ** *p* < 0.01, *** *p* < 0.001, **** *p* < 0.0001, 2-way ANOVA.

We next tested whether the protective effect of lipids on ferroptosis could be observed in vivo. To ask this question, ovarian cancer cells were injected into the peritoneal cavity of immune-compromised mice, a system we have previously used to model intraperitoneal metastases of ovarian cancer, and in which direct intraperitoneal injection of erastin inhibits tumor growth [7]. Since murine as well as human adipocytes can protect ovarian cancer cells from ferroptosis in vitro (Figure 1), we reasoned that blocking uptake of lipids by inhibiting CD36 would reduce the cytoprotective effect of adipocytes in this experimental setting, thus enhancing the sensitivity of ovarian tumors to ferroptosis in vivo. To test this hypothesis, we first confirmed that sulfo-N-succinimidyl oleate (SSO), an inhibitor of the lipid transporter CD36 [43], inhibited the protective effect of ACM on ferroptosis induced by erastin in vitro (Supplementary Materials Figure S7). We then treated tumor-bearing mice with SSO for 15 days before intraperitoneal challenge with the ferroptosis inducer erastin. Mice were sacrificed 15 days later and tumor number and mass measured. As shown in Figure 11 and Supplementary Materials Figure S8, erastin significantly reduced tumor burden, reducing both tumor number and mass. As anticipated from previous reports demonstrating that inhibition of CD36 reduces tumor burden in ovarian cancer xenografts [35], SSO alone also reduced tumor burden. However, reduction in tumor burden in mice subjected to combined treatment with erastin and SSO was significantly greater than the tumor number and mass reduction observed in mice treated with either agent alone (*p* ≤ 0.001) and led to almost complete tumor elimination under these experimental conditions (Figure 11). Thus, blockade of CD36-mediated lipid transfer sensitizes tumor cells to ferroptosis in vivo.

## 4. Discussion

Ovarian cancer differs from most malignancies in that metastases are generally disseminated by local invasion within the peritoneal cavity rather than through hematogenous spread. Within the peritoneum, the primary site of metastases is the omentum, an adipocyte-rich tissue that facilitates and supports metastases through the release of bioactive molecules [44] such as adipokines and long-chain fatty acids. Fatty acids released by adipocytes are taken up by cancer cells, promoting rapid tumor growth through beta oxidation [26] as well as facilitating drug resistance [15].

In this work we describe an additional role for adipocytes in ovarian cancer: protection from ferroptotic cell death. Ferroptosis is a process of cell death that is intimately connected to lipid metabolism. In particular, oxidized polyunsaturated fatty acids esterified to phospholipids are believed to drive ferroptotic cell death [1,2,3]. Accordingly, a major class of ferroptosis inducers, exemplified by RSL3, act by disabling GPX4, an enzyme that reduces fatty acids in phospholipids. Similarly, enzymes that lower levels of polyunsaturated fatty acids, such as SCD1, protect cells from ferroptosis [41].

The intimate relationship between ovarian cancers and adipocytes led us to query whether adipocytes might influence ferroptosis of ovarian cancer cells through modulation of lipid metabolism. We report here that co-culture of ovarian cancer cells and adipocytes results in the transfer of lipids from adipocytes to ovarian cancer cells (Figure 6), and that this reduces the cytotoxic effect of ferroptosis inducers on ovarian cancer cells (Figure 1 and Figure 2). Consistent with the unique dependence of ferroptosis on lipid metabolism, adipocytes did not protect ovarian cancer cells from apoptotic or necrotic cell death (Figure 3).

Lipidomic profiling of cells with and without exposure to ACM showed a strong separation by cell type, indicating that cell identity is a strong driver of global lipid composition. Within each cell type, ACM induced structured shifts in lipid abundance, reflecting coordinated changes across distinct clusters of lipids (Figure 7). These patterns suggest the presence of co-regulated lipid sets that respond systematically to ACM treatment. Importantly, the magnitude and direction of these treatment effects differed between cell types, indicating that ACM responses are context-dependent rather than uniform. Nevertheless, some consistent changes were observed, including notable decreases in PUFA-containing PEs and increases in cholesterol esters, both consistent with a reduction in ferroptosis.

The ability of cells with stem-like properties (“cancer stem cells”) to seed ovarian cancer metastases has been suggested [45]. We therefore included a model of ovarian cancer stem cells in our studies [7,18]. We observed that these cells are susceptible to ferroptosis, and that, like HGSOC cells, they were protected from ferroptosis inducers by ACM in vitro (Figure 2). Since spheroid formation is believed to be involved in the dissemination of ovarian cancer within the peritoneum, we also tested whether exposure to ACM would protect ovarian cancer spheroids from a challenge with ferroptosis inducers. Similar to cells grown in 2D, these 3D cultures were also protected from RSL3 by ACM (Figure 4). Importantly, when these cells were injected in mice, treatment with SSO, an agent that inhibits CD36 and blocks lipid uptake, potentiated the anti-tumor effect of the ferroptosis inducer erastin (Figure 11). Thus, the contribution of adipocytes to ferroptosis resistance extends to ovarian cancer cells with stem-like properties, suggesting that combining ferroptosis inducers with agents that block lipid delivery may be effective in reducing ovarian cancer metastatic spread.

The protective effect of ACM on ferroptosis allowed us to dissect molecular components responsible for this effect. Our work points to a major contribution of lipid transfer from adipocytes to cancer cells in the protection from ferroptosis. First, protection can be conferred by ACM and does not require direct cell contact (Figure 2). Second, protection is diminished by lipid depletion of the conditioned medium as well as by inhibition of fatty acid uptake pathways in ovarian cancer cells (Figure 6). Third, exposure to ACM not only significantly changes the lipid composition of ovarian cancer cells but increases their lipid droplet content (Figure 7 and Figure 8). The increase in lipid droplets is likely to be an important contributor to the cytoprotective effect of ACM, since inhibition of lipid droplet formation by two independent methods—blockade of DGAT 1/2 or inhibition of SCD1—both attenuated the cytoprotective effect of ACM (Figure 9 and Figure 10). Overall, these findings suggest that adipocytes protect ovarian cancer cells from ferroptosis by donating lipids and increasing lipid droplet formation.

Several limitations of our study should be acknowledged. First, our work utilized human adipocytes differentiated from subcutaneous preadipocytes rather than cancer-associated adipocytes (CAA). CAA exhibit several characteristics, including overexpression of inflammatory cytokines, proteases, and morphological features that distinguish them from non-cancer-associated adipocytes and contribute to their effect on ovarian cancer cell biology [46]. Thus, CAA may influence the response to ferroptosis inducers in ways not captured in our analysis. Additional experiments using systems that more completely recapitulate the omental microenvironment, including the presence of CAA as well as cancer-associated fibroblasts and other cell types, will be required to confirm our results as well as to fully contextualize the contribution of adipocytes to ferroptosis in vivo. Second, ACM contains both factors produced by adipocytes and factors depleted by adipocytes; thus, in addition to the protection conferred by adipocyte-derived lipids (Figure 6, Figure 9, Figure 10 and Figure 11), depletion of selected factors may also contribute to the protective effect of ACM on cancer cells. Third, many of our studies utilized cells grown in 2D culture, a model that incompletely captures 3D tumor cell biology [47]. Nonetheless, our observations that 3D ovarian cancer spheroids are protected from ferroptosis by the adipocyte-conditioned medium (Figure 4), and that blockade of lipid transfer influences the response of tumors to ferroptosis in vivo (Figure 11), support the relevance of our observations to tumor biology in vivo.

Our work does not rule out the existence of additional mechanisms by which adipocytes protect tumor cells from ferroptosis. While this manuscript was in preparation, it was reported that adipocytes protect colorectal tumor cells from ferroptosis by elevating 3-hydroxykynurenine, a tryptophan metabolite that modifies NCOA4 and suppresses ferritinophagy to increase levels of ferritin, an iron storage protein [48]. Increased ferritin in turn exerts a protective effect on ferroptosis by limiting the iron available to catalyze lipid peroxidation. We also noted that exposure to ACM increased ferritin (Supplementary Materials Figure S9), although we observed an increase in ferritin mRNA as well as protein, suggesting an additional or alternative mechanism for the increase in ferritin under our experimental conditions. Overall, based on the extensive diminution in the cytoprotective effect of ACM following blockade of lipid delivery both in vitro (Figure 6) and in vivo (Figure 11), we conclude that modulation of lipid metabolism is a major mechanism by which adipocytes protect ovarian cancer cells from ferroptosis.

Our observations are consistent with a previous report that lipid droplet formation induced by cell cycle arrest confers ferroptosis resistance [38]. Since ferroptosis is driven by peroxidation of polyunsaturated fatty acids, these authors concluded that lipid droplet formation suppresses ferroptosis by reducing the pool of polyunsaturated fatty acids available for oxidation by sequestering them in triacylglycerols. Consistent with this interpretation, we noted a significant decrease in multiple triglyceride species containing PUFAs following exposure of either OVCAR4 or FTT cells to ACM. However, our analysis was qualitative and not quantitative, so the impact of lipids provided by adipocytes on overall levels of PUFAs for participation in ferroptosis remains undetermined. Whether PUFA availability or other mechanisms underly the ability of increased lipid droplets to provide the protection from ferroptosis that we observed in this study will require further investigation. In either case, disabling the protective effect of lipid transfer from adipocytes to ovarian cancer cells may be key to the effective deployment of ferroptosis inducers in the treatment of ovarian cancer.

## 5. Conclusions

Our findings identify adipocytes as critical regulators of ferroptosis resistance in ovarian cancer. We demonstrate that adipocyte-derived lipids protect ovarian cancer cells, including stem-like populations and multicellular spheroids, from ferroptotic cell death induced by mechanistically distinct ferroptosis inducers. This protective effect is mediated largely through lipid transfer and uptake, resulting in extensive remodeling of cellular lipid composition and increased accumulation of lipid droplets. In vivo, inhibition of CD36-mediated lipid uptake augmented the anti-tumor effect of the ferroptosis inducer erastin, leading to near-complete tumor elimination under these experimental conditions.

These results establish a direct mechanistic link between the adipocyte-rich ovarian cancer microenvironment and ferroptosis resistance, highlighting metabolic crosstalk as an important determinant of therapeutic response. More broadly, our study suggests that the efficacy of ferroptosis-based therapies in ovarian cancer may depend not only on intrinsic tumor cell susceptibility, but also on the metabolic support provided by the tumor microenvironment. Targeting adipocyte-to-tumor lipid transfer pathways, lipid storage mechanisms, or associated metabolic adaptations may represent a promising strategy to enhance ferroptosis sensitivity and overcome metastatic persistence and therapeutic resistance in ovarian cancer.

## Data Availability

The original contributions presented in this study are included in the article and Supplementary Materials. Further inquiries can be directed to the corresponding author.

## Supplementary Materials

The following supporting information can be downloaded at https://www.mdpi.com/article/10.3390/biom16101418/s1, Figure S1: siRNA knock-down reduces CD36 and FABP4 mRNA. OVCAR4 and FTT cells were transfected using CD36 siRNA or FABP4 siRNA for 72 h, and knock-down efficiency was measured using q-PCR. Means and std dev of 3 replicate samples: one of 3 independent experiments; Figure S2: Treatment with erastin, SSO, or erastin plus SSO does not affect overall mouse body weight. Mice were weighed at the initiation of treatment and every five days thereafter; Figure S3: Exposure to ACM reduces lipid peroxidation in cells treated with RSL3. A. ACM or control medium was applied to OVCAR4 cells for 48 h prior to treatment with vehicle (Veh) or RSL3 for 2 h; cells were stained with C11-BODIPY and analyzed by flow cytometry. B. Quantification of C11-BODIPY mean relative fluorescence; means and SD of data from 2 independent experiments with ≥3 replicas per experiment, * *p* < 1.1 × 10^−8^; Figure S4: ACM does not affect proliferation. OVCAR4 and FT-T cells were exposed to control or ACM and cell number was assessed in triplicate cultures after 24, 48 or 72 h with trypan-blue; Figure S5: FTT cells cultured as spheroids retain sensitivity to erastin and RSL3. FTT cells were allowed to form spheroids for 24 h before treatment with erastin or RSL3 at doses ranging from 1–20 µM for 24 h and assessment of cell viability using ATP based Celltiter Glo. Means and std dev of 3 to 8 replicate samples; one of 3 independent experiments is shown. Comparison to untreated control, * *p* < 0.04; Figure S6: Lipid depletion does not affect cell viability. OVCAR4 and FT-T cells were plated for 24 h before treatment with control medium, ACM, lipid depleted control medium or lipid depleted ACM for 48 h. Cell viability was assessed using calcein-AM. Means and std dev of triplicate cultures; Figure S7: Inhibition of CD36 with SSO blocks the cytoprotective effect of ACM in vitro. FT-T cells were incubated in control medium, ACM, or ACM containing the CD36 inhibitor SSO for 24 h, followed by treatment with the indicated concentrations of erastin for 24 h. Means and std dev of 4 replicate samples; one of 3 independent expts, * *p* < 1.9 × 10^−6^; Figure S8: Representative tumor images. A. The image shows an array of 15 mL tubes containing tumors collected from mice in 4 treatment groups. Each tube contains pooled tumors harvested from an individual mouse (8 mice/group for vehicle and SSO; 7 mice/group for Era and Era+SSO). After harvest, tumors were counted and weighed in aggregate prior to formalin fixation Dashed red boxes indicate the sample used in illustrations 8B and 8C. B. The image shows individual tumors from a representative mouse in each treatment group. C. The image shows H&E-stained sections of formalin-fixed paraffin-embedded tumor tissue from one mouse in each treatment group. Histology is consistent with high grade tumors (200× magnification); Figure S9: Exposure to ACM induces ferritin mRNA and protein. OVCAR4 and FT-T cells were exposed to control or ACM for 48 h; Cells were harvested and analyzed by western blot or q-PCR; Table S1: Lipid species with significant changes in both FTT and OVCAR4 cells.

## Abbreviations

The following abbreviations are used in this manuscript:

ACM	Adipocyte-conditioned medium
CoQ	Coenzyme Q
CPT1	Carnitine palmitoyltransferase 1
DGAT	Diacylglycerol acyltransferase
GPX4	Glutathione peroxidase 4
GSH	Reduced glutathione
GSSG	Glutathione disulfide
HGSOC	High-grade serous ovarian cancer
PE	Phosphatidylethanolamine
PUFA	Polyunsaturated fatty acid
SCD1	Stearoyl-CoA desaturase
SSO	Sulfo-N-succinimidyl oleate
